# Gauging public perceptions of military and police roles in US domestic pandemic response during COVID-19

**DOI:** 10.3389/fpubh.2025.1569263

**Published:** 2025-06-18

**Authors:** Evan Warren, Congruo Wang, Megan Rhodes, David P. Polatty, Adam C. Levine

**Affiliations:** Center for Human Rights and Humanitarian Studies, Watson Institute for International and Public Affairs, Brown University, Providence, RI, United States

**Keywords:** military, police, National Guard, COVID-19, pandemic, Civ-Mil, response, perceptions

## Abstract

**Introduction:**

Militaries and police forces have been increasingly deployed in response to humanitarian crises and public health emergencies. Existing studies have largely been concentrated around international interventions, overlooking US domestic contexts and the perceptions of those receiving aid.

**Methods:**

In recognition of these gaps, this research involved a survey of 1,500 Americans to understand opinions toward the utilization of the US military and local law enforcement as COVID-19 domestic pandemic responders at an unprecedented scale.

**Results:**

A majority were complimentary of and comfortable with these armed actors' role in the response and supportive of involvement in future crises, with the military regarded more favorably than police. Trust in civilians, the military, and police is found to be role-based; favorability was inherently tied to the nature of services provided, whether healthcare, logistics, or enforcement-related. Perceptions were also strongly linked to one's vaccination status, political party affiliation, ideology, age, and gender. Underlying trust in civilian providers was evident, but often did not preclude one from favorable views of the military and law enforcement.

**Conclusion:**

Ultimately, these results have implications on domestic policy in future national crisis scenarios and highlight the need for further research exploring if sentiment holds steady beyond the realm of public health and pandemics.

## 1 Introduction

On January 20, 2020, the U.S. Centers for Disease Control and Prevention (CDC) confirmed that the first laboratory-tested case of the 2019 Novel Coronavirus (COVID-19) had been recorded in the United States. In the immediate days following, the first request was made of the Department of Defense (DOD) to aid in domestic pandemic response: opening March Air Reserve Base as a quarantine facility for U.S. Department of State officials returning from Wuhan, China, the epicenter of the outbreak. This appeal from the Department of Health and Human Services was approved just 9 days after the confirmation of the first case, marking the beginning of the US military contribution to domestic COVID-19 relief efforts (1). Initially, aid was limited to such “evacuee installations” and “funneling sites,” which expanded in number as larger quantities of at-risk travelers returned home. However, the scope of US military response would rapidly evolve and swell in subsequent months as millions of Americans contracted the virus.

In the 2 years following, as many as one million members of the armed services—drawn from the Army, Air Force, Navy, and Coast Guard—were activated to support the American healthcare system (2). Additionally, over 47,000 members of the National Guard were summoned to aid in both federal and state-level responses (3, 4). Classified, along with law enforcement, as “emergency responders” critical to healthcare efforts, these soldiers fulfilled a variety of roles (5). Personnel were sent to provide direct medical care in overwhelmed civilian hospitals, build overflow facilities, establish field hospitals in major cities, disseminate information and supplies, run testing centers, and provide administrative support, even driving school buses and teaching in schools (6, 7). Military coordination also extended into the strategic and logistical realm. The defense establishment lent its expertise in rapid contracting to procure supplies, manufacturing assistance, supply chain management, crisis action planning, and vaccine development through Operation Warp Speed (7). Few aspects of the American pandemic response were devoid of any military influence or involvement.

Academic interest in the relationship between civil society and the military has been well-documented and theorized in the decades since World War II, especially in the United States under the transition to an all-volunteer force. In recent years, as global humanitarian crises have increased in cost and number, a related field has emerged—humanitarian-military relations (HMR)—which examines the relationships between civilian responders and armed actors during crisis response.^1^ While this literature has presented typologies for HMR in the public health emergency context, it generally makes assumptions about the relationship between crisis-affected community members and armed actors. Few studies have directly examined these public perceptions of armed actor intervention in humanitarian or public health settings, to include the military, law enforcement, and non-state armed groups.

Given the extent of US military and law enforcement involvement in domestic pandemic response in 2020, the global prevalence of armed actors in humanitarian settings, and the lack of relevant research on the sentiment of crisis-affected individuals toward military responders, there exists a noticeable gap in the extent of current HMR literature. This research attempts to fill that gap by exploring how the American public viewed the use of the military, National Guard, and law enforcement in the domestic COVID-19 response. Findings from the comprehensive survey of 1,500 individuals, documenting levels of comfort and perceived appropriateness of uniformed service members and police fulfilling non-traditional roles, can inform the scope of future involvement in public health emergencies and create a foundation for further research.

## 2 Methods

A cross-sectional survey was conducted to evaluate public perceptions of military and law enforcement involvement during the COVID-19 pandemic in the United States. The study aimed to capture a nationally representative sample of 1,500 respondents through stratified random sampling. Demographic strata included age, gender, race/ethnicity, political affiliation, and vaccination status, based on U.S. Census benchmarks.

The survey was administered online via YouGov, a secure platform adhering to strict privacy and confidentiality standards. Informed consent was obtained electronically before participation. The study protocol was reviewed and granted a human subjects research exemption by the Institutional Review Board at Brown University (Protocol #STUDY00000279).

### 2.1 Survey instrument and variables of interest

The survey instrument was developed through a comprehensive process drawing from existing literature in public perception research and validated measurement scales. It was then pilot tested on a group of five key informants with expert knowledge on the use of military and law enforcement in public health emergencies and revised based on their feedback before being administered to survey participants. Finally, the survey instrument was reviewed by YouGov staff for clarity and consistency with similar surveys administered to its representative panel.

This research incorporated a multifaceted approach to data collection, utilizing Likert Scale measurements to assess comfort levels and perceptions of military and law enforcement involvement. Multiple-choice questions were designed to capture preferences for healthcare worker types and gather detailed demographic information. The structured open-ended response was designed to provide qualitative depth, allowing participants to elaborate on their quantitative ratings and provide nuanced perspectives on military and law enforcement involvement during the pandemic, thereby complementing the statistical data with rich, contextual narrative insights.

The study operationalized both dependent and independent variables to provide a comprehensive understanding of pandemic-related perceptions. Dependent variables included participants' comfort levels with institutional involvement, preferences for healthcare workers, and overall perceptions of military and law enforcement contributions during the pandemic. Terms presented to survey respondents, such as “comfort” with armed actors, “quality” of response, and “support” for future involvement were not explicitly defined, allowing individuals to interpret them subjectively. These terms are commonly utilized in this manner throughout similar survey instruments that gauge public opinion across a variety of other issue areas.

Independent variables encompassed a wide range of demographic characteristics, including age (categorized into 18–29, 30–44, 45–59, 60+ years), gender, race/ethnicity, political affiliation, vaccination status, and previous voting behavior. This comprehensive approach allowed for a nuanced exploration of how demographic factors influence institutional perceptions.

### 2.2 Analytical framework and statistical methods

Statistical analyses were conducted using computational tools, including Stata/BE version 17.0 (StataCorp, College Station, TX) and Python version 3.8.20 (Python Software Foundation, Wilmington, DE), with a predetermined significance level of *p* < 0.05. Given the complexity of the survey design and the need for robust interpretation, sophisticated techniques were employed to account for potential biases and design effects. These included data preparation strategies such as data cleaning to address missing or inconsistent values, the application of survey weights to ensure representativeness of the national population, and bias mitigation approaches to correct for non-response and selection bias.

The analytical methods included a diverse range of statistical techniques. Descriptive statistics, including means, medians, frequencies, and percentages, provided an initial understanding of the data and highlighted key demographic patterns. Inferential statistical analyses were employed to explore associations and differences across demographic and attitudinal variables. For categorical data, chi-squared tests were used to assess independence between variables. For non-parametric comparisons, the Kruskal–Wallis *H*-Test and Wilcoxon Rank-Sum Test were applied to evaluate differences in perceptions across demographic subgroups, such as age, vaccination status, and political affiliation.

Advanced statistical modeling techniques were applied to examine the determinants of public perceptions, offering a comprehensive framework for understanding complex associations. Logistic regression and multinomial logistic regression were utilized to analyze the relationships between categorical dependent variables and multiple independent predictors, enabling the identification of significant factors influencing public attitudes and preferences. Together, these methods provided a robust analytical approach, uncovering nuanced relationships and generating reliable conclusions regarding public perceptions of military and law enforcement roles during the COVID-19 pandemic. Regression analyses utilized baselines categories collected by YouGov to ensure a balanced survey population. Existing literature does not indicate a relationship between these characteristics—among them, age, gender, race, ethnicity, geographic location, political leanings, previous voting behavior, and vaccination status—and opinions on the use of armed actors in pandemic response. Thus, all of these categories were included as covariates in regression modeling.

All analyses were conducted with attention to the underlying assumptions of the statistical tests and models, and sensitivity analyses were performed to validate the robustness of the findings. This multi-faceted approach ensured that the results were reliable, interpretable, and grounded in the complexity of the survey data.

### 2.3 Qualitative methods

Additionally, a quasi-applied thematic analysis was implemented to complement the statistical methods that were central to this research. Of the 37 questions posed to respondents, only one was appropriate for a qualitative approach: a catch-all, open-ended question to end the survey, requesting that respondents leave any comments, insight, or feedback regarding the military/National Guard and law enforcements' role in the national response to COVID-19. A majority of participants provided some degree of substantive comment beyond “none” or “N/A,” allowing for all submissions to be combed through and coded. In grouping these responses, five key themes emerged that were applicable to many comments left behind. In order of the quantity of applicable responses received, these themes were: (1) Comfort with Military Response, (2) Concerns with Law Enforcement, (3) Rationale for Preferring Neither, (4) Rationale for Choosing Civilians over Military, and (5) Comfort with Civilian Providers. After sorting relevant comments into broad categories, quotes that illustrated the essence of the prevailing sentiment among each group were extracted to weave into the final manuscript and provide additional context for quantitative insights.

## 3 Results

### 3.1 Participant demographics and observations

The survey that this research is built upon randomly sampled from the American population to create a representative cross-section that accurately reflects the views of the country at-large. To ensure that the results and conclusions that follow are externally valid, the demographic markers associated with participants were verified to be within realistic ranges compared to population-wide estimates. Ultimately, this was found to be true, with those demographic attributes of our 1,500-person sample presented below as Table 1.

**Table 1:** Descriptive statistics of demographic attributes.

**Attribute**	**Category/statistic**	**Percentage (%)**	**Count (*n*)**
Age (continuous)	Mean	–	47.52
	25th percentile	–	32
	Median (50th)	–	47
	75th percentile	–	62
	SD	–	17.43
Gender	Male	46.87	703
	Female	53.13	797
Race	White	66.40	996
	Black	13.67	205
	Hispanic	10.47	157
	Asian	3.67	55
	Native American	1.27	19
	Two or more races	2.33	35
	Other	1.73	26
	Middle Eastern	0.47	7
3-point party ID	Democrat	36.27	544
	Republican	28.33	435
	Independent	24.73	371
	Other	4.33	65
	Not sure	6.33	95
Region	Northeast	18.20	273
	Midwest	19.60	294
	South	38.73	581
	West	23.47	352
COVID vaccination	Fully vaccinated (booster)	44.00	660
	Fully vaccinated (no booster)	23.13	347
	Partially vaccinated	6.67	100
	Not vaccinated (want soon)	1.00	15
	Not vaccinated (waiting info)	3.13	47
	Don't want vaccination	17.67	266
Ideology (5-scale)	Very liberal	10.47	157
	Liberal	16.60	249
	Moderate	34.47	517
	Conservative	20.20	303
	Very conservative	10.80	162
	Not sure	7.47	112
2016 vote	Hillary Clinton	25.47	382
	Donald Trump	29.87	448
	Gary Johnson	1.93	29
	Jill Stein	1.27	19
	Evan McMullin	0.27	4
	Other	1.07	16
	Did not vote	40.13	602
2020 vote	Joe Biden	34.27	514
	Donald Trump	32.67	490
	Jo Jorgensen	0.47	7
	Howie Hawkins	0.27	4
	Other	0.60	9
	Did not vote	31.73	476

For the questions contained within the survey to be relevant to respondents, these individuals would require some means of previous exposure to law enforcement or military actors at any point during the pandemic, whether firsthand or through other secondary avenues. Survey participants were directly asked about personal observation, the highest bar of relevance. Nearly half of all participants personally observed some degree of military or National Guard involvement. Of those who recalled personal experience with uniformed military during the pandemic, the most prevalent response, at 17%, was that the respondent was uncertain of service branch affiliation. The two service branches observed at rates over twice that of others were the National Guard, by 16% of respondents, and the Army, by 14% of respondents. Irrespective of personal observations, the National Guard was also believed by respondents to be the most involved in the COVID-19 response, and by a sizable margin. Police were personally observed by 30% of Americans in activities pertaining to the COVID-19 response—this total exceeds any individual branch of the military but is less than the 46% of Americans that observed any degree of military relief efforts. Roughly the same share of participants—just over a third—agreed that law enforcement was “heavily” involved in the response efforts.

**Figure 1:**
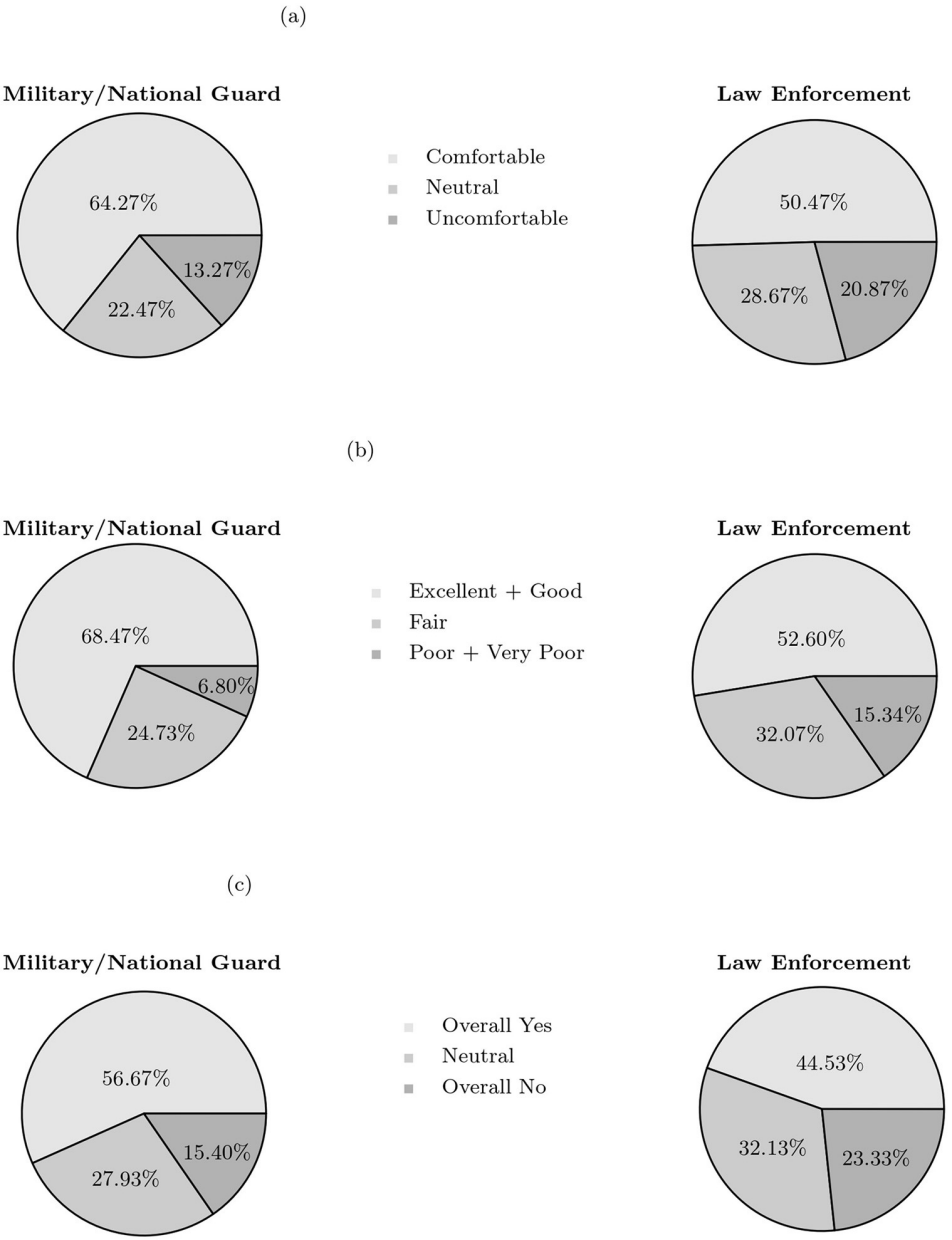
Comparison of public perceptions regarding military and law enforcement involvement during the COVID-19 response. **(a)** Comfort level with involvement. Survey question: “How comfortable are you with the thought of having the US military/National Guard/Law Enforcement involved in a pandemic response?”. **(b)** Contribution rating. Survey question: “How would you rate the military/National Guard/Law Enforcement's overall contribution to the COVID-19 pandemic response in the US?”. **(c)** Support for future increased involvement. Survey question: “If the U.S. faced another national crisis, would you support increased military/National Guard/Law Enforcement involvement based on their role during the COVID-19 pandemic?”.

The consensus among respondents was that military involvement was warranted in relief efforts, irrespective of one's degree of observation of the military response. The majority of respondents agreed that the military and National Guard were utilized appropriately, whereas 13% disagreed to some degree with the necessity of the military response. Similar to sentiment toward the military, the majority of respondents believed that police involvement was a necessary element of the overall pandemic response. Still, military necessity scored nearly 20 percentage points higher than law enforcement necessity. While central themes themselves, these perspectives were explored in greater depth throughout the rest of the survey. Specifically, this survey went on to dissect the affected population's degree of comfort with military and law enforcement actors, how these individuals rated the quality of military and law enforcement features of the pandemic response, and if they would support future involvement of either entity in emergency settings. The raw data from these three core themes is displayed below as Figure 1, with corresponding results and analyses detailed in the sections that follow.

### 3.2 Comfort with armed actor pandemic response

As depicted in Figure 1a, study participants were asked about their level of comfort with military and National Guard members' and law enforcement members' involvement in a pandemic response, respectively. The left pie chart illustrates that those surveyed were largely receptive to military involvement. Over 60% of respondents reported being comfortable with the thought of a military-aided crisis response. Roughly 22% were neutral, with 13% indicating some degree of discomfort. A majority of respondents were also comfortable with the concept of law enforcement being involved in pandemic response, illustrated in the pie chart on the right of Figure 1a. That being said, comparing military to law enforcement, the magnitude of those comfortable shrunk by nearly 14 percentage points, and the magnitude of those uncomfortable increased by more than seven percentage points.

Given the mismatch in measured comfort between military responders and law enforcement responders, survey results were further analyzed to understand if certain demographic characteristics consistently contributed to such differences in sentiment. First, a Kruskal–Wallis *H*-Test was conducted, capable of distinguishing statistically significant differences in responses across groups, and in this case, age groups: 18–39 years old, 40–64 years old, and over 64 years old. Median comfort with the military COVID-19 response was not significantly different among those belonging to different age groups. In contrast, median comfort with the law enforcement response did vary slightly by age group. Most comfortable with police involvement were adults over the age of 64, followed by those in the youngest cohort, ages 18–39. The least level of comfort was observed among the middle-aged group, spanning ages 40–64. These results are displayed in Supplementary Table S1.

Another tool utilized to understand the relationship between demographic characteristics and survey responses was multivariate regression, which can control for the influence of extraneous factors to isolate the impact of a certain variable of interest. This tool adds context to the most important questions examined in this survey. Comfort with the military providing services during COVID-19 decreases with belonging to a racial group other than “white,” not receiving a COVID vaccination, affiliating with political parties other than the Democratic party, and self-identifying as ideologically conservative.

Similar variables are observed as influencing law enforcement comfort. The magnitude of the effect associated with political parties and COVID vaccination status slightly decreased from the military to law enforcement comfort models, retaining their significance. Race and ideology drop their significance, and age emerges as a factor in slightly decreasing one's comfort with police. In Supplementary Table S2, the full regression output is provided, with statistically significant findings pertaining to military and law enforcement comfort demarcated with asterisks. More asterisks indicate a smaller likelihood that the relationship was due to random chance. Of note, these findings do not change when utilizing different reference groups for each independent demographic variable.

Ultimately, these regression models demonstrate that the largest decreases in comfort are tied to one's vaccination status: those lacking a full vaccination are over 57% less likely to be comfortable with a military response to a pandemic, as indicated by the corresponding decrease in the odds ratio column of Supplementary Table S2. Lacking a vaccination produces a 51% likelihood of being uncomfortable with a police response. Of similar significance but smaller magnitude of effect is political party identification. Non-democrats are over 33% less likely to be comfortable with a military response and over 31% less likely to be comfortable with a police response. Age, race, and ideology produce their own effects of smaller magnitude, and while still statistically significant, are not consistent across military and law enforcement models.

### 3.3 Rating of armed actor contribution to pandemic response

Next, respondents were asked to evaluate the individual contribution from the military and National Guard and law enforcement agents toward overall pandemic response efforts. While respondents had the ability to choose responses including “excellent,” “good,” “fair,” “poor,” and “very poor,” the positive and negative responses were grouped together to increase compatibility with statistical modeling. The results from this survey question are depicted in Figure 1b, with military rating displayed in the left pie chart and law enforcement rating on the right.

The overall military and National Guard response was highly regarded by the American public. Only 6.8% of respondents gave the armed services poor marks regarding their contribution to COVID-19 relief efforts. “Fair” and “excellent” were descriptors that both attained roughly a quarter of responses, with the characterization of “good” achieving a plurality at just under 43%. Altogether, positive responses constituted a clear majority, comprising over 68% of responses.

Gaps between perceptions of military and law enforcement actors were more distinct in the quality metric. A slight majority felt positively about the police contribution to pandemic efforts, totaling 52.6% of responses. Far more participants were ambivalent, with the “fair” rating garnering just over 32%. The number of respondents regarding the contribution of police negatively more than doubled compared to that of the US military.

Kruskal–Wallis *H*-Tests demonstrated that, similar to the comfort models, perceptions of the quality of both the military and police response also were slightly different across age groups. In general, the youngest cohort tended to rate armed actor responses the highest. While the middle-aged group averaged the lowest rating, it was only nudged by the over-64 cohort by a small margin. This finding is also included within Supplementary Table S1.

Multivariate regression results for perceptions of quality mirror those of perceptions of comfort among the American population. Where military response rating is the dependent variable, lacking a full vaccination predicts a 54.9% decrease in likelihood of positive evaluation. Membership in a political party other than Democratic reduces the likelihood of positive rating by nearly 25%. For law enforcement, these same two variables are significant, with vaccination status playing a slightly smaller role and party affiliation a slightly larger role in odds of negative rating. In this model, age and ideology also possess statistical significance, where increasing age slightly decreases the odds of rating the police response positively and possessing an ideology other than “liberal” increasing the likelihood of a respondent providing a favorable rating. The full extent of this regression is displayed in Supplementary Table S3.

### 3.4 Support for future involvement in domestic crises

Following inquiries of comfort and quality was a question that asked respondents to draw upon the experiences of COVID-19 to evaluate if military or law enforcement actors should possess a role in future national crises. The results from this question are illustrated in Figure 1c. Whereas 57% of Americans would again be pro-military involvement, only 45% would support an increased law enforcement presence. Similarly, 15% are against increased military involvement, vs. 23% against increased police involvement. A larger share of participants was neutral toward the police than the military and National Guard.

As with the two prior questions, multivariate regressions were conducted to determine the factors that influenced public support for future military involvement of the military and law enforcement. The full output can be found in the Supplementary Table S3. Largely similar to comfort and quality models, COVID-19 vaccination status and political party affiliation produce the most significant and highest magnitude decreases in public support. Lacking a vaccination produces an almost 60% decrease in the odds of supporting future involvement of the military, and almost 50% for police. Similarly, identifying as a partisan other than Democrat predicts a 34% decrease in support for future involvement of the military and 31% decrease in support for future involvement of police. Race is only a significant factor for future military involvement, with those identifying as other than white predicted to have slightly lower levels of support. Finally, age is significant in both the military and police regressions, but the magnitude of effect is negligible.

### 3.5 Coordination and collaboration

**Figure 2:**
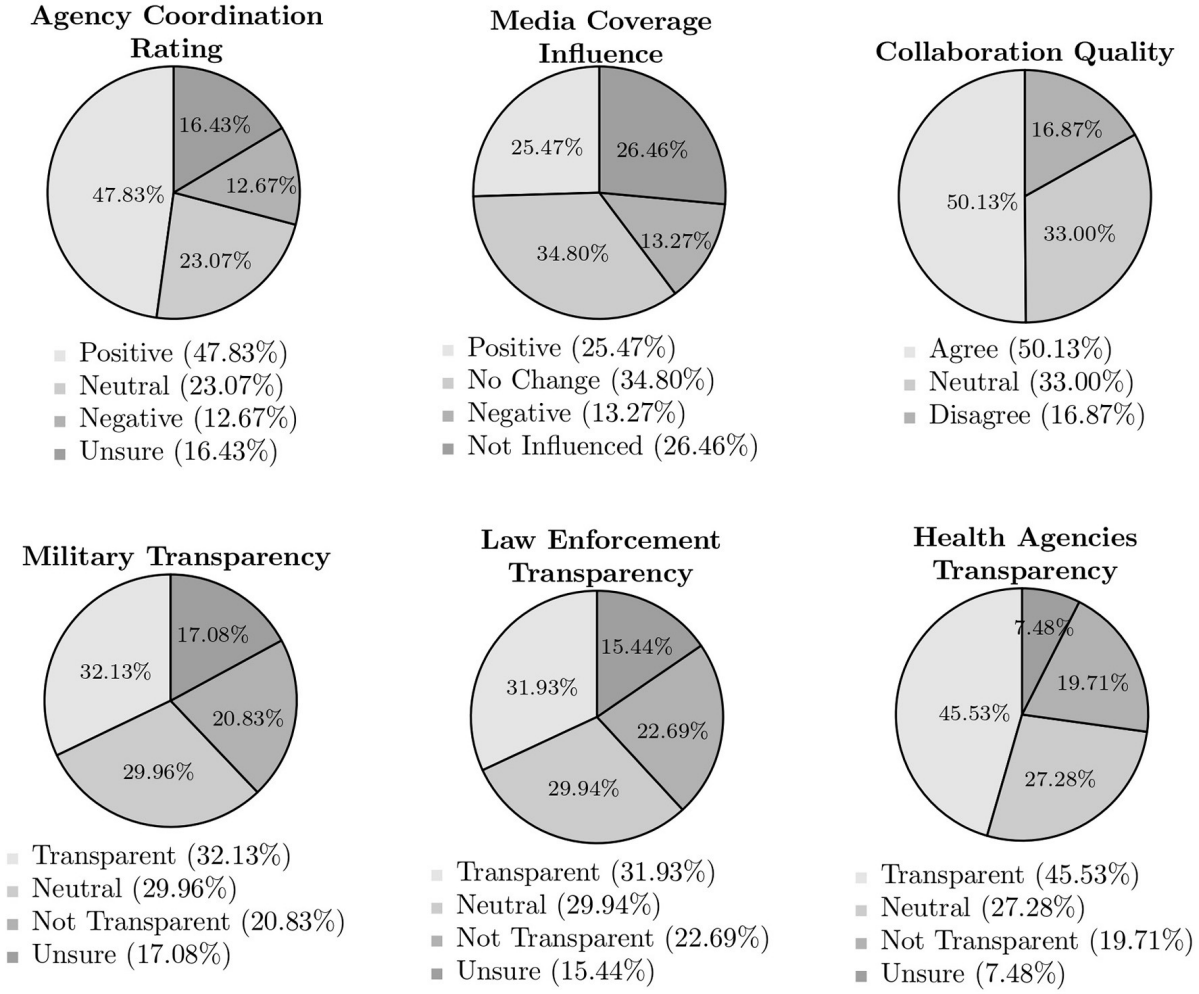
Military, law enforcement, and health agencies communication and coordination during COVID-19 pandemic. Percentages are rounded to two decimal places. “Positive” combines “Excellent” and “Good” responses; “Negative” combines “Poor” and “Very Poor” responses; “Transparent” combines “Very Transparent” and “Transparent” responses; “Not Transparent” combines “Not Transparent” and “Not at all Transparent” responses; “Agree” combines “Strongly Agree” and “Agree” responses; “Disagree” combines “Disagree” and “Strongly Disagree” responses.

After examining sentiment toward the military and law enforcement individually, this survey briefly explored more overarching themes that were shared between these entities and the civilian health sector during the pandemic. Respondents' evaluations of these themes are illustrated below in Figure 2. The top row of pie charts address perceptions of coordination between the military, law enforcement, and civilian healthcare workers; if the collaboration between these entities improved the quality of response provided to Americans; and the role of media coverage in shaping one's views of coordination and collaboration. The bottom row compares participants' assessments of transparency of each entity–military, law enforcement, and civilian health agencies–during the pandemic.

The most prevalent sentiment expressed by respondents regarding interoperability during the pandemic was one of a positive nature. The highest share of positive responses was garnered by the question that asked if collaboration between all involved groups–both armed and civilian–improved the overall COVID-19 response, earning just over 50%. Slightly fewer, 47.8%, believed that overall coordination between the military, law enforcement, and civilians was effective. That said, the number of positive responses halved to 25.5% when evaluating the role of media coverage in influencing perceptions of these entities' capacity to collaborate, though most reporting not experiencing a change of opinion or not being influenced. Across these three questions, negative sentiment earned roughly the same share, ranging from just under 13 to just below 17% of responses, and was consistently surpassed by indifference or positivity.

Questions of transparency elicited a near-mirror image for both types of armed responder, creating a clear contrast with opinions toward civilian health agencies. Civilians were found to be transparent by about 13% more of respondents than their military, National Guard, and law enforcement counterparts during the pandemic. Similarly, more than double the number of participants expressed uncertainty in assessing transparency when dealing with armed actors vs. civilian healthcare providers. Relatively constant across all three entities were the proportion of those who identified themselves as neutral or found the entity in question to not be transparent. Even so, the civilian providers still edged the armed actors in fewest “Not Transparent” evaluations at 19.7%, and law enforcement emerged at the top end with 22.7%.

### 3.6 Provider preference in theoretical pandemic scenarios

The final type of question facing survey respondents was prospective and theoretical, deviating from the largely reflective prompts that preceded it. With this final series, individual categorizations of providers were pitted against one another in plausible pandemic scenarios, with participants tasked with expressing their ultimate preference. The first question isolated the military and National Guard, offering the choice between a uniformed or a civilian healthcare provider to administer a COVID-19 vaccination, all else equal. Civilian healthcare workers were preferred by 25% of respondents, vice uniformed military service members at 13%. That being said, the majority-−51%—indicated no preference between the two. Nearly 11% of respondents would choose neither the civilian nor the military COVID-19 vaccine administrator.

Considering that police often did not fulfill healthcare roles like civilian and military responders, the second scenario was altered to better facilitate the inclusion of law enforcement agents. Participants were asked about which of the three types of providers, civilian, military, or police, would be preferred to relay information about pandemic guidelines. Police garnered the lowest total, at 7%, with the military roughly double that at 13%. Almost 40% of Americans would prefer the civilian healthcare worker, but 30% were fine with any of the three. A nearly identical share of respondents as in the vaccine hypothetical would be uncomfortable with any of the three types of responders providing pandemic guideline information.

As with previous questions, further statistical analyses were conducted on these results to understand what demographic factors may motivate participant responses. To determine the range of demographic characteristics that played a significant role, this analysis began with a chi-squared test, detailed below as Table 2. Asterisks indicate a relationship between the provider one selected in the two hypothetical survey questions of interest and variables related to their personal background that is not due to random chance alone.

**Table 2:** Demographic determinants of COVID-19 vaccine distribution and guideline communication preferences.

**Covariate**	**COVID-19 vaccine distributors**			**Agents informing pandemic guidelines**		
	**Chi-square (**χ^2^**)**	**df**	*p* **-value**	**Chi-square (**χ^2^**)**	**df**	*p* **-value**
Gender	18.51	3	0.000^***^	25.8349	4	0.000^***^
Race	25.31	21	0.234	58.9039	28	0.001^**^
Vaccination status (2022)	260.06	18	0.000^***^	310.9423	24	0.000^***^
Region	23.37	9	0.005^**^	23.7233	12	0.022^*^
3-point party ID (pid3)	83.39	12	0.000^***^	119.1591	16	0.000^***^
Ideology (ideo5)	83.90	15	0.000^***^	100.4789	20	0.000^***^
Presidential vote post 2016	77.02	18	0.000^***^	100.3705	24	0.000^***^
Presidential vote post 2020	65.25	15	0.000^***^	118.7001	20	0.000^***^

^*^p < 0.05, ^**^p < 0.01, ^***^p < 0.001.

As seen in Table 2, the lone variable lacking a statistically significant relationship to choice of provider is the race of the respondent, and this is only true in one of the two questions–the vaccine administration hypothetical that excludes the choice of law enforcement agents. Across both questions, all other demographic variables were indicated to possess some degree of influence by this statistical model, to include the gender of respondent, their vaccination status, the region of the country they reside in, their political party affiliation, their political ideology, and the vote they cast for president in the 2016 and 2020 election cycles. That being said, the chi-squared test alone does not offer any further insight into how these demographics shaped preferences, which requires additional modeling.

In order to gather further insights from the two hypothetical survey questions, this research utilized two multinomial logistic regressions, with the results presented below. Table 3 contains the output related to the first question of vaccine-related services, which excludes police. Table 4 details the results related to the second question, which includes all categorizations of responder as a provider of pandemic-related public health guidelines. Each corresponding value represents a relative risk ratio, with asterisks indicating a degree of statistical significance. For each variable, the condition listed in parentheses is the reference group. This means that, for instance, men are 1.594 times as likely, or almost 60% more likely, as women to prefer a civilian healthcare worker vs. having no preference between the two, and to a statistically significant degree (which, spatially, is the upper left-most significant finding presented in Table 3).

**Table 3:** Multinomial logistic regression results: provider preferences for COVID-19 vaccine, treatment, or testing services.

**Variable**	**Civilian**		**Military**		**Neither**	
	**RRR**	**SE**	**RRR**	**SE**	**RRR**	**SE**
Age	0.964	0.023	0.926^*^	0.030	1.043	0.037
Age^2^	1.000	0.002	1.000	0.003	1.000	0.004
Gender (female)	1.594^***^	0.206	1.497^*^	0.251	1.259	0.236
Race (White)	0.991	0.048	1.018	0.062	1.046	0.064
COVID vaccination^a^	1.153	0.172	1.105	0.209	6.768^***^	1.391
Region (South)	0.907	0.049	1.076	0.074	0.910	0.072
Party ID (Democrat)	0.974	0.069	0.683^***^	0.067	1.213	0.127
Ideology (Liberal)	0.994	0.079	1.175	0.119	1.592^***^	0.184
Vote 2016 (Clinton)	0.976	0.069	0.958	0.086	0.901	0.105
Vote 2020 (Biden)	0.950	0.067	1.052	0.091	1.127	0.132
Constant	1.904	1.107	3.181	2.337	0.013^***^	0.011

^*^p < 0.05, ^**^p < 0.01, ^***^p < 0.001.

RRR, relative risk ratio.

^a^Reference group: fully vaccinated with booster and fully vaccinated without booster.

Base outcome is “No Preference” category.

**Table 4:** Multinomial logistic regression results: provider preferences for pandemic guidelines information.

**Variable**	**Law enforcement**		**Military**		**Civilian**		**Uncomfortable**	
	**RRR**	**SE**	**RRR**	**SE**	**RRR**	**SE**	**RRR**	**SE**
Age	0.963	0.045	1.013	0.034	0.998	0.023	1.021	0.037
Age^2^	0.999	0.001	1.000	0.003	1.000	0.002	1.000	0.003
Gender (female)	2.106^***^	0.483	1.819^***^	0.319	1.201	0.155	1.693^**^	0.331
Race (white)	0.953	0.085	1.000	0.067	1.012	0.049	1.114	0.069
COVID vaccination^a^	1.576	0.387	1.078	0.212	0.910	0.136	5.744^***^	1.222
Region (South)	1.262^*^	0.119	1.032	0.076	1.113	0.060	0.958	0.079
Party ID (Democrat)	0.689^**^	0.093	0.780^*^	0.078	1.110	0.079	1.456^***^	0.159
Ideology (Liberal)	1.063	0.147	0.970	0.103	0.759^**^	0.060	1.159	0.140
Vote 2016 (Clinton)	0.763^*^	0.095	0.918	0.086	0.884	0.062	0.923	0.109
Vote 2020 (Biden)	1.066	0.127	1.051	0.097	0.965	0.069	0.997	0.118
Constant	1.423	1.535	0.772	0.622	2.076	1.188	0.032^***^	0.030

^*^p < 0.05, ^**^p < 0.01, ^***^p < 0.001.

RRR, relative risk ratio.

^a^Reference group: fully vaccinated with booster and fully vaccinated without booster.

Base outcome is “Comfortable with all options” category.

In addition to the gender-related finding described in the preceding paragraph as an example, Table 3 displays five other statistically significant findings pertaining to the vaccination hypothetical, all compared to a base outcome of lacking any preference at all. The finding of the largest magnitude is that those lacking a full COVID-19 vaccination are nearly seven times more likely than those with vaccinations to prefer neither civilian nor military healthcare workers to provide a vaccination. The other factor resulting in a statistically significant increase in preference for neither provider is possessing an ideology other than “liberal,” producing a 59.2% jump in probability.

Examining factors associated with preference for military healthcare providers, political party affiliation is the most statistically significant predictor. Belonging to a party other than the Democratic makes one 31.7% less likely to prefer a military provider compared to a Democratic participant. Additionally, male respondents were almost 50% more likely to prefer a military vaccine administrator than females. Finally, being of older age resulted in a small decrease in the odds of preferring a military provider compared to younger participants.

Within Table 4 are 10 statistically significant results pertaining to the likelihood of certain demographic characteristics to predict participant comfort with a specific group of responder to relay pandemic public health guidelines, compared to being comfortable with all three groups of providers. Gender again plays a role, with males over two times more likely than females to be comfortable with law enforcement and 1.8 times more likely than females to be comfortable with a uniformed service member. Slightly less statistically significant is the relationship between gender and discomfort with all providers, where males are 69% more likely to be uncomfortable than females. Of highest statistical significance and magnitude is the finding that lacking a full COVID vaccination makes a respondent 5.7 times more likely to be uncomfortable with any provider in this scenario, compared to those with full vaccinations.

Partisans other than Democrats were 31% less likely to select the police, 22% less likely to select the military, but over 45% more likely to be uncomfortable with all pandemic guideline conveyors. The one statistically significant factor that decreased the likelihood of choosing a civilian responder was possessing ideology other than liberal, resulting in a predicted 24% decrease. Finally, belonging to a geographic region outside of the South increased the odds one would be comfortable with a law enforcement agent, while voting for a candidate other than Hillary Clinton in the 2016 election decreased one's odds of possessing that same preference.

### 3.7 Qualitative context

The final question included in the survey that is the foundation of this research was a free response question, allowing participants to share any final thoughts or observations of the military, National Guard, and law enforcement response to the domestic COVID-19 pandemic. Responses clustered around a number of central themes, and illustrative quotes emblematic of the central tenets of each theme are included within this section to add context demonstrating the common viewpoints shared by participants that had potential to shape responses.

The modal sentiment shared by participants in this portion of the survey involved expressions that indicated broad acceptance and support of military responders. Respondents characterized the military as the organization that “stepped up” to “do an excellent job” when the “medical profession was over-burdened.” Some trusted the military because of familiarity or prior service, but even some who were admittedly “not ‘pro-military' found the presence of uniformed troops assisting in healthcare roles “reassuring.” Respondents noted that the military was “uniquely equipped with special skills” that are tailored to emergencies, frequently singling out logistics and supply as marked strengths. However, many were simply impressed at how coordinated, professional, and responsive the military efforts they observed were.

The second most common theme involved concerns with law enforcement agents as pandemic responders. Despite a majority having rated the overall police contribution “excellent,” “good,” or “fair,” there was a higher incidence of dissatisfaction expressed in open-ended responses when compared to the military. On one hand, some participants believed that, as a large federal organization, the military was often better coordinated and efficient than local-level law enforcement, which were characterized as “inconsistent.” A difference in roles was also noted, with the military more involved in healthcare and police more involved in enforcement of rules and regulations, the latter being an inherently less popular role. Others voiced consternation with observations of law enforcement encouraging anti-vaccine sentiment, including not wearing masks and choosing not to enforce regulations. More common were comments about police being generally incompetent and poorly trained, often accompanied by statements like “the police are not to be trusted.” Recent high-profile instances of abuse and racial tensions were cited as evidence for these claims.

While not the perception held by the majority, a very outspoken minority of participants voiced a strong averseness to vaccination efforts and government response as a whole. Data from this question alone cannot prove that the “neither” option available in hypothetical scenarios was selected by most due to anti-vaccination beliefs, but it did yield an abundance of qualitative data to support the anti-vaccination notion. By far, the most prevalent comment was that the pandemic was a “farce,” a “hoax,” or a “scam” in which the military was complicit with a government and healthcare system that, at best, “overreacted,” and at worst, “enforced illegal policies.” Civilians were associated with “Big Pharma” and an untrustworthy healthcare system and military actors as the government's means of controlling citizen behavior. Some simply objected on the grounds that the vaccine was unnecessary or unsafe, which has clear implications on a question about receiving an inoculation, as the first hypothetical scenario was presented.

Finally, a theme that was indisputably present but collected the fewest number of responses was related to the ultimate preference of civilians over any flavor of armed actor. Participants voiced that “only civilian personnel…should be involved in any medical decisions,” and that “medical professionals and respected scientists should be trusted the most.” This group made clear that their perspectives were event-based, noting pandemics are distinct from other civil unrest that calls for law enforcement and the National Guard or emphasizing that the military should solely be utilized in event of a foreign threat. Others voiced concerns with power dynamics, characterizing armed actors as “intimidating,” “angry,” or “aggressive.” Those in this cohort tended to think that resources would be better invested by “supporting actual healthcare professionals” instead of using them to integrate institutions with other primary mission sets in the overall pandemic response.

## 4 Discussion

### 4.1 Existing literature and frameworks

#### 4.1.1 National and international frameworks

In principle, only dire circumstances warrant military involvement in humanitarian environments. The United Nations Office for the Coordination of Humanitarian Affairs has issued two guiding documents for military involvement in peacetime disaster response: the 2007 Oslo Guidelines and the 2018 Recommended Practices for Effective Humanitarian Civil-Military Coordination of Foreign Military Assets.^2^ Established within are two key concepts: distinction and last resort. The former underscores the importance of distinguishing between civilian and military actors, lest the lines between neutral humanitarian responders and armed combatants be blurred (8–10). The latter addresses when it is appropriate to augment a humanitarian response with military resources—both documents note that circumstances should be exceptional, with armed forces responding to fill gaps that lack a comparable civilian alternative (10, 11). Further guidance states that the military responders should at all times remain under civilian control, and that relief be administered unarmed, in national military uniform (11).

While the United Nations guidance is thorough, it contains a crucial caveat: both documents are oriented toward external military intervention in foreign disasters. The stipulations above do not necessarily apply to national militaries responding domestically (11). Humanitarians have historically translated such principles into domestic settings, but there is a marked lack of individualized guidance, especially regarding epidemics and pandemics (12). Neither set of guidelines bar a nation from deploying national militaries as first responders, as they omit any references to interventions by a state to rescue its own citizens (13, 14). Additionally, neither document explicitly addresses responses to emerging infectious diseases (12).

As a result, domestic deployments of armed actors in humanitarian crisis scenarios vary widely, as do national-level policy frameworks. Many countries do possess statutes regarding when military capabilities may be activated in response to domestic needs (15). Though these policies are not aligned with a higher standard, the military is frequently institutionalized by nations as a first responder (12). That being said, very few national-level policies explicitly or clearly include for health emergencies (15). In the United States, the Federal Emergency Management Authority policy mirrors that of the United Nations' 2007 and 2018 guidance (7). The US military is to be used as a last resort in emergency settings, in recognition of the high costs associated with employing military capabilities. Western government foreign policy has shifted to increasingly permit the deployment of military assets in health sector support, and with intrastate responses presumably requiring a lower bar for clearance, this suggests a similar trend domestically (16).

#### 4.1.2 Trends in military responses to humanitarian emergencies

Globally, incidences of military involvement in humanitarian settings are on the rise. The intersection between humanitarians and militaries is not a new phenomenon—the embedding of relief organizations into armed forces is a part of a broader pattern that can be traced to the First and Second World Wars (17, 18). Some humanitarians critique that the continuation of this trend, where relief organizations insert themselves into peacekeeping environments, has resulted in the militarization of aid (9, 17). However, in recent years, the scale of international humanitarian assistance needs has grown with each successive year, creating demands that militaries are often called upon to fill (10, 12). Military assets are now regularly being used to provide unique capabilities in aid settings and across a variety of sectors, domestically and abroad (7, 13, 19). Given these developments, military responses, especially in domestic settings, are likely to become the norm, rather than the exception, in coming years (12).

The increasing prevalence of military involvement in crisis scenarios was underscored in the world's response to the COVID-19 pandemic. The majority of nations mobilized some degree of armed actor involvement in their domestic responses to the virus (7). Previous health crises have been put into security terms, but the level of military intervention was heightened beyond what has been observed in the prior decade, even considering intervention in foreign outbreaks (20, 21). There is some debate as to whether government and military-led campaigns against epidemics are a novel development, but most scholars agree that COVID-19 placed militaries in new and unfamiliar roles (18, 21). Nevertheless, the pandemic has been framed as a pivotal event that has entrenched armed actors as routine participants in the health realm going forward, leading some to predict a fundamental restructuring of HMR or extreme securitization of future domestic disaster situations (19, 22).

#### 4.1.3 Law enforcement interventions in public health

By definition, law enforcement agents are first responders, which makes their regular presence in emergency settings no surprise. Yet, lacking a specific healthcare element comparable to their military counterparts, police are not a seamless fit into public health responses. On one hand, law enforcement is naturally oriented toward the same mission as public health workers: protecting citizens from ill health, injury, or unnatural and untimely death (23). Even before the COVID-19 pandemic, recent years had seen an unprecedented growth in collaboration between the two sectors, especially in welfare states and high-resource settings (24). Nevertheless, observers still note an appreciable divide. Cooperation between law enforcement and public health authorities does occur on an *ad hoc* basis–sporadically, and often, reluctantly–but mutual suspicion and hostility remain (23). Establishing cooperation between these two spheres is an ongoing, drawn-out, vacillating process, with some arguing it is neither beneficial nor desirable (25, 26).

Historically, major health crises have thrust the policing and public health realms together, providing the most visible examples of collaboration. Certain types of public health emergencies always require enforcement roles (27). Less frequently, these are events like bioterrorism. The Anthrax attacks of 2001 saw cooperation between public health experts and law enforcement investigators (26). More often, cooperation occurs under the premise of an epidemic or pandemic, where police elements are tasked with enforcing and explaining public health measures on the front lines. Law enforcement agents have assumed this role in many of the major outbreaks and pandemics of the past century, including the H1N1 Flu of 1918, Severe Acute Respiratory Syndrome (SARS) in 2002, the H1N1 Flu of 2009, and most recently, COVID-19 (28).

#### 4.1.4 Armed actor involvement in the domestic COVID-19 response

The United States military provided a litany of services during the COVID-19 pandemic, drawing on capacity and expertise that was otherwise scarce. Due to its size and cache of resources, the military is capable of rapidly deploying large quantities of personnel, equipment, and supplies (10, 20). This strength was leveraged in the United States. All 50 states, Washington DC, and nearly every territory used National Guard soldiers liberally as frontline workers, filling roles ranging from vaccine administration to meal distribution and other social services (18, 29). Often, military and law enforcement were a complementary force, responding in addition to civilians in public health and social service settings; the sheer scale of the pandemic demanded action from every state and municipality, which drove the need for infusing personnel from the National Guard, local police forces, and the various branches of the military.

Furthermore, the US military possesses an inherent proficiency in areas relevant to pandemic response. The American military medicine establishment is at the forefront of emerging infections and is highly adept at developing vaccines to combat them (7). The defense stockpile was rich with personal protective equipment, rapidly distributed via resilient logistical networks (18, 21). Security expertise was leveraged to control borders and enforce stay-at-home orders (18). Evidently, the top-down nature of military operations is effective at cutting through red tape to quickly respond in adverse environments, though theorists warn it may also serve to diminish the role and perspectives of civilian partners and civil society (20).

Also involved in pandemic response efforts, American law enforcement agents were delegated an unprecedented scope of public health-related roles. Early in the pandemic, in the absence of a vaccine, police compelled and monitored adherence to behavioral interventions, including physical distancing in public spaces, mask mandates, stay-in-place orders, and establishment closures (27, 30). That being said, law enforcement did not forfeit their ever-expanding role as social service providers. Despite not physically administering healthcare, police were still involved in the provision of resources for local communities, from disseminating information on pandemic guidance to filling gaps in various public service roles (28). Although not explicitly equipped to serve in a public health capacity, police were generally recognized as a critical arm of pandemic response.

#### 4.1.5 Public perception of military interventions in crisis and pandemic response

Trends indicating widespread military utilization in crisis scenarios have not yet produced substantive research into the resulting perceptions among the populations being served. Numerous studies acknowledge the lack of documentation and analysis of such perspectives, as well as their importance to understanding the future of civil-military interactions (7, 8, 12, 21, 31). Relevant studies that do exist are sparse and tend to examine international contexts, with even fewer centered around public health emergencies (7, 21, 32). Generally, HMR-oriented literature assumes that the relationship between community members and militants is negative or highly fraught, rooted in studies occurring outside the realm of COVID-19 but with crisis-affected populations in international settings (8, 21).

While the literature examining causes and consequences of domestic military operations is robust, there is little consideration given to how the public responds to such events in the United States (33, 34). One of the lone inquiries, a poll of Americans regarding support for the use of law enforcement authorities, the National Guard, and the military in various domestic responses, found that generally, the public was more skeptical of military vs. police interventions, but that approval increased for military and National Guard responders in a disease outbreak scenario, with support for law enforcement stagnant (31).

Domestic public opinion analysis has generally occurred in the context of foreign intervention, attempting to identify the factors that incentivize and disincentivize the American public to back a military operation abroad. The perception of a mission's legitimacy is a primary determinant of support (35, 36). The public is also likely to evaluate the totality of costs—including human and financial—associated with an intervention and weigh those against any benefits (37–39). Consistent with the importance of political parties in America is their role in shaping perceptions. Both the party of the sitting president and the degree of consensus between party elites influence widespread support of an international operation (40–42). Finally, operations with higher expectation of success are generally more popular than those with lower odds (35, 43). There is no guarantee that these same factors influence perceptions of domestic responses but they can be utilized as a comparative tool.

Broadly, in the United States, the military is alone in the trust and prestige it garners from the general population, a phenomenon that was solidified after the September 11, 2001 terror attacks (44–46), though has declined somewhat in recent years (44). One of the few studies that has recorded public sentiment toward military intervention during COVID-19 occurred in New York State, finding that public responses to aid from servicemembers was generally very positive (29). However, the same study noted that public expectations could be managed better through a more unified messaging campaign. Another study of attitudes toward the US military's COVID-19 response found that the public was often initially mistrustful of the military presence but warmed to the response as the military took measures to address concerns and increase familiarity (7). A different, wider examination of public sentiment toward domestic military action around the world during the pandemic found no correlation between trust and usage (22). A greater breadth of pandemic-centered research is required to advance any of the aforementioned conclusions.

#### 4.1.6 Public perception of law enforcement interventions in pandemic response

This pandemic research involved a survey that first asked respondents about their perceptions toward the military and National Guard during COVID-19, later delivering the same set of questions regarding law enforcement. In recent years, the factors that determine the legitimacy of law enforcement responses have been well-documented. Legitimacy is increased when police behave in ways that conform with procedure and perceived fairness (47–49). Police support wanes when forces are used in visible and aggressive ways to maintain order or when the police appear more militarized (50–52). Evidence demonstrates that police forces have, in fact, become more militarized, which may have implications on questions that explore the distinction between the armed forces and law enforcement in America (53, 54).

Applying these concepts to law enforcement utilization throughout the pandemic, there is reason to believe that the American public would be similarly divided in their attitudes toward police response. Across the globe, a regular feature of law enforcement involvement was the enforcement of various public health measures, but this same characteristic served as a basis to challenge the legitimacy of policing (27). Where citizens disagreed with the regulations enacted, their frustrations tended to spill over onto those upholding them. Evidence also shows that citizens' observations of police responses and perceptions of effectiveness drive broader feelings of trust, confidence, and quality of ensuing community relations (30). Complicating this picture during COVID-19 were protests against policing sparked by high-profile uses of force against racial minorities, making it difficult to isolate perceptions regarding pandemic response because of how the events became intertwined (27). Ultimately, the literature exploring the relationship between pandemics, public health, and the police is limited, further underscoring the importance of this survey as a means to fill gaps in existing research.

### 4.2 Implications of survey results

#### 4.2.1 Relevance

Responses collected from survey participants offer a range of insights that fill in a number of the gaps identified in the existing literature on armed actor responses to humanitarian and public health crises. First, underscoring the importance of this research is the surprising degree to which respondents personally observed armed actor involvement in pandemic relief efforts. An implicit assumption in conducting this study was that the American public had some degree of exposure to armed actor response. Without it, many of the questions asked would not have produced informative data. The military did mobilize an unprecedented number of troops to assist, but not all were in public-facing roles–this fact did not limit participant observation. Additionally, survey responses were collected in early 2024–after the official close of the pandemic–and some recollections of military and police involvement may have faded as mass media and the public consciousness shifted elsewhere. To have half of the representative sample with individual encounters with uniformed personnel during COVID-19 is an astoundingly high mark. Many qualitative responses underscored that these encounters were firsthand and meaningful. Respondents noted the strengths of individual branches with comments like, “the National Guard members I saw did an excellent job,” and referenced the specific settings in which they encountered these responders. One participant recounted: “[I] received my first two vaccines at a field operation…it was organized and efficient. [The military personnel] were more polite and respectful than the medical personnel.” Participants also widely perceived police officers to have a significant role in pandemic response, despite the roles that law enforcement assumed during the pandemic potentially appearing similar to the duties they generally fulfill. These findings support the concept of conducting such research in the first place and bolster the relevance of the other questions contained within the survey.

#### 4.2.2 Relationship to existing research

Current voids in HMR literature are centered around affected population sentiment. The few existing studies in this arena examine the experience of crisis-affected populations when military actors are introduced to humanitarian settings, almost always in an international context. Even fewer analyze pandemic settings and US popular sentiment toward internal military and law enforcement nontraditional responses. Thus, any indication of public comfort with armed actors involved in a domestic setting constitutes a key finding.

In international contexts, armed actors are typically not given trust or benefit of the doubt in crisis response. Relationships with affected populations are presumed to often be highly fraught (8). Specific case studies support the conclusion that, at best, the prevailing public sentiment is that of ambivalence, with large variability in opinion toward military involvement in humanitarian response (12). Even when intentions are positive, cited barriers to mutual understanding are public misconceptions about the role of the military, cultural differences between armed actors and locals, and awareness of human rights violations previously precipitated or capable of being precipitated by combatants (21). That being said, these results consistently demonstrate that the majority of American citizens were comfortable with or neutral to the way that military and law enforcement actors responded. Within this study, respondents tended to be more deferential than affected populations previously observed in international settings. Even with high levels of comfort across the board, a hierarchy did emerge, with military actors generally favored over law enforcement. Uniformed service members garnered recognition from respondents for their ability to mobilize quickly in emergencies, amass a great deal of manpower, and apply vast expertise in the logistical and medical realms. These strengths were also noted in open-ended responses, where participants both drew upon previous familiarity with military effectiveness and their positive observations from within healthcare settings. Police generally did not receive the same amount of praise in survey or open-ended responses. Many noted that they “specifically trust police less” as they “have nothing to do with a pandemic.” Whereas international studies find no correlation between affected population trust and usage of military and law enforcement actors during COVID-19 (22), this study does register a correlation between usage and positive perception in the United States.

These results reinforce the findings from a similar study conducted within New York State that indicated the general public was very receptive to aid administered by the National Guard and other branches of the armed forces (29). This previous work also noted the deleterious effects of imprecise information, misreporting, and contradictory material on public opinion. While such elements were undoubtedly present on a national scale, evidenced in the number of our survey participants espousing common conspiracy theories as a justification for their responses, there is also reason to believe that effective coordination and communication influenced many respondents' perceptions of the military as well. A plurality of the participants in this study felt positively about media portrayals and transparency, with a majority in approval of coordination between the military, police, and other civilian institutions. A majority also felt comfortable with military aid and the quality of response. This research supports that military actors were generally held in high regard during COVID-19 response in America and the importance of unified, effective messaging to such sentiment.

Furthermore, these results indicate that a plurality would still opt for civilian providers if all else was held equal. Participants did agree that military elements could replace civilian healthcare workers–unlike law enforcement, which was generally not favored over civilians–but most appreciated the ability of armed actors to augment a response, vice lead the response. A majority believed that civilians should take the predominant role in COVID-19 relief efforts, though this did not preclude military or police involvement. Civilians prevailed in both hypothetical scenarios offering a choice of provider and assessments of transparency. This theme also garnered a notable degree of qualitative support, as some remarked that “medical expertise should be left to medical staff and researchers” and that “it is better to manage crises similar to [COVID-19] by civilian institutions, not military ones.” Nevertheless, comfort with armed actors and trust in civilians are not mutually exclusive; these results prove that both were present throughout the COVID-19 pandemic in America.

One may not have expected law enforcement to fare worse in rating compared to the military, as law enforcement officers are embedded in communities and involved in local service provision. The disparities in satisfaction earned by law enforcement vs. other actors may be related to two fundamental differences in the roles played throughout the pandemic. First, police generally were not involved in healthcare efforts at all. Unlike the military, law enforcement agencies do not possess a large medical apparatus that can be substituted for the same types of roles and services as the civilian healthcare system. Participants observed this, stating things like “law enforcement shouldn't have anything to do with healthcare.” Instead, police officers were largely serving in enforcement and oversight roles, which may have made them a target of ire. Whereas military members were observed directly administering health-related aid, police were often compelled to confront and correct members of society. Often, the rules they were tasked with enforcing were unpopular to begin with, which is supported by statements left by respondents such as “police were forced to enforce idiotic and capricious rules and quasi-laws.” Second, the pandemic coincided with large anti-policing protests and sentiment that headlined the news and spread rapidly across the nation. Law enforcement agents were clashing with rioters at the same time that they were fulfilling COVID-19 relief roles, which may have shaped some individuals' perceptions. One respondent noted that, “me being a Black man, I don't trust police in any form for anything.” These realities likely influenced the military being favored by a majority to contribute in future national crises, but the police receiving only a plurality of support.

One recent study by Blankshain et al. (31) found that the American public is generally more skeptical of the military than police or the National Guard. Though the study utilized as the foundation of this paper largely did not distinguish between the National Guard and other branches of the armed forces, assuming that most respondents would associate all uniformed service members as military, the findings of this survey are contrary to that of Blankshain et al. (31). Across the board, the military earned higher marks from respondents than law enforcement within this survey. Yet, Blankshain et al. (31) concede that National Guard and military favorability would increase in an outbreak scenario, a finding that is supported by this research. Scholars have previously noted that observations of police responses and subsequent evaluations of effectiveness drive feelings of trust and confidence (30). Concerns with law enforcement were a prevailing qualitative theme in comments left by respondents. Of military, civilian, and police responders, police generally were rated the least effective. These findings corroborate that disaster context and roles fulfilled can shape public opinion, with law enforcement drawing ire for being largely stuck with enforcement tasks in an environment where the military and civilians were better suited to meet healthcare needs.

#### 4.2.3 Context-specific findings

A particular factor unique to this public health emergency was the scope of denialism and anti-vaccination sentiment present amongst the American population. This presents a marked challenge to the survey provided to participants. The survey questions present the pandemic as factual and real, which a group of respondents objected to altogether and expressed resentment toward. In different emergency settings, it is entirely possible that the denialist and anti-vaccination groups would possess separate views of government-affiliated actors, such as military service members and police, than those presented here.

Given this unique characteristic of COVID-19 in America, any question in the survey involving mention of vaccination received a similar level of negative responses. A small portion of participants appeared to respond in opposition to any mention of government response or vaccine administration to combat the pandemic, against the totality of intervention efforts. Both quantitative and qualitative data confirmed that a subset of the population was skeptical of the virus itself and the government's role in the pandemic. An outspoken minority made this skepticism clear with statements such as: “I believe the vaccine was a hoax and the police and military involvement was complicit with the government.” It is possible that, had the nature of the crisis been less controversial, these individuals would have been warmer to the idea of government-affiliated armed actors assisting in relief efforts. However, given that the military and law enforcement are government actors themselves, this group of respondents saw little distinction between such entities.

Thus, the persistent role of vaccination status as a strong predictor of participant perceptions conforms with the degree of controversy that ultimately engulfed the inoculation movement. Across nearly every metric of interest in this research–from perceptions of comfort, to rating of response, to opinions regarding future involvement, and even the ways in which respondents approached the hypothetical questions–the lack of a vaccination was a reliable predictor of one possessing negative sentiment toward pandemic responders, which even included civilian healthcare workers. This observation is not simply a function of politics or other demographics–the multivariable regressions conducted in this study also included party affiliation, ideology, age, and gender as controls, which are all generally correlated with vaccination status. The remaining effect of vaccination status could be expected to be smaller after accounting for their influence, but even so, was consistently the most statistically significant and of the largest magnitude of any independent variable. Even when the reference group is altered to the lowest level of aggregation, where only those possessing a full vaccine with booster are the basis for comparison, vaccination status still influences outcomes, as shown in the alternate models included in the Supplementary material.

Relatedly, given the degree of political polarization in America and the pervasiveness of partisan identification, unsurprising is the role that politics played in respondent's answers to survey questions. Political ideology and partisanship were also among the most persistent variables involved in shaping perceptions, evidenced by statistically significant and high-magnitude results across many iterations of regression models. Patterns of responses formed along party lines–those who self-identified as Democrats tended to support military and law enforcement responses, whereas those who self-identified as Republican were less supportive. On one hand, it would be reasonable to have expected the opposite relationship. Very progressive movements, like defunding the police, had been mounted against law enforcement agents within this timeframe. Similarly, conservatives have been linked with “backing the blue” and exhibiting high levels of support for American troops. That being said, Democrats are also generally comfortable with greater government intervention, whereas Republicans tend to be more skeptical. The latter viewpoint was only exacerbated by anti-vaccination sentiment and conspiracy theories spread throughout the pandemic. Previous studies of public perception of foreign intervention signal the importance of party affiliation (40–42). This research finds that partisan affiliation is similarly important in this domestic pandemic environment.

Although closely linked to political parties and ideology in America, age was not a major determinant of comfort with military or law enforcement actors in multivariate regression. This is likely due to regression models controlling for political leanings, which drive most age-related variance. Small differences were observed in the univariable analyses between the youngest, middle-aged, and oldest age groups, with the youngest cohort generally the most accepting and highest-approving of military and law enforcement responders. Middle-aged and older participants rating the response of armed actors slightly lower could be a result of relative unfamiliarity with the concept of armed actor interventions in crisis settings, which have increased in frequency during the modern era, or diverging sets of intra-generational values, with older participants possessing more traditional views. Race was also notably absent as a determinant of perceptions regarding military and law enforcement response. After controlling for factors such as political leanings, age, vaccination status, and voting behavior, variation across the racial groups and ethnicities represented within this study were negligible and did not reach statistical significance.

Previous analyses of international contexts acknowledge that perceptions often vary widely amongst a given population, influenced by the content of the role being performed, with those least consistent with expectations being the most controversial (8, 15). Thus, the range of factors that shape public perception in the United States and the various lines that emerge between demographic groups conform with expectations rooted in preceding work. While in this research, striations emerge primarily along political boundaries, more so than in studies of less polarized nations and international emergency settings, constant is the importance of the role being performed by an actor matching the expectations and norms of the affected population.

#### 4.2.4 Implications on public health policy

In a democratic society like that of the United States, a premium is placed on understanding and influencing public perception. This is especially true about health considerations, as they carry a particularly high salience—pandemic scenarios create a fear of being blamed for doing too little to protect public health, affecting decisions made by elected officials (55, 56). Ultimately, voter perceptions matter given that political leaders are single-minded seekers of re-election, which means that said leaders are predisposed to disruptive policies to create the impression of responsiveness to public health (55). Pressure on democratically-elected leaders is high to preserve human life and wellbeing, but a key consideration is choosing a strategy that resonates with the electorate who is receiving the aid.

Thus, among the most critical policy implications of this research involves whether or not the American public would be supportive of increased involvement from military or law enforcement in future crises. The data from this survey points to the public being largely supportive. In a public health emergency, the military would be favored over law enforcement, but the public was satisfied with both actors and how they coordinated their response. Future research should explore public perception in other types of domestic and international crises, as it may alter which group receives the most favorability. Ultimately, even in public health crises, there is still a desire for medical experts to lead the response. Informed medical expertise is important to Americans, even if the administration of aid is performed by those without it.

### 4.3 Limitations

Though statistically rigorous, these findings are limited, in part, due to the approach utilized to obtain them. Participants were primarily asked to utilize Likert Scale responses to characterize various assessments of pandemic responders and armed actors. Whereas this scale increases accessibility, flexibility of responses, and ease of interpretation, it also possesses its own drawbacks. When responding utilizing Likert Scales, individuals may have the tendency to agree with statements regardless of their true perceptions or to avoid extreme responses and gravitate toward the middle of the scale. Fixed response options can also limit participants' ability to express nuanced opinions and are liable to subjectivity, with interpretations of their meaning varying between individuals. As such, the ordinal choices made by respondents cannot be assumed to be spaced at equal intervals in statistical modeling. Despite being pilot tested on key informants and reviewed by YouGov for clarity and consistency with similar surveys, the psychometric properties of the instrument were not otherwise evaluated before its administration to study participants. Also of note, while YouGov utilizes a balanced panel representative of the broader American population, there are a range of individual characteristics not assessed by the platform. Those unmeasured characteristics may be differently distributed among panel members than the public, which would potentially produce less generalizable study results.

The survey of 1,500 Americans was cross-sectional, representing the opinions of the sample at only the moment that responses were received. This research alone cannot demonstrate how perceptions have evolved or been influenced by factors over a period of time. Relatedly, this survey was administered in January of 2024, 8 months after the official end of the COVID-19 public health emergency in the US. In the United States, cases spiked at various points from 2020 to 2022, with most disaster declarations and armed actor mobilization present at the beginning of the pandemic. Thus, survey respondents were years removed from the height of observed pandemic response, potentially introducing an element of recall bias into our results. This bias occurs when participants systemically err in retrieving accurate and complete recollections of events and experiences from the past. Respondents were relied upon to accurately remember elements of a major phenomenon which may have been colored by events between their observations and enrollment in this study, and it is possible that findings may have been different if this study was administered closer to the onset of COVID-19.

Including an open-ended question in the survey design allowed for qualitative features to be integrated into the analysis. However, the qualitative portion of the study was not rooted in a true grounded theory framework; rather, it incorporated elements of applied thematic analysis to make inferences about the common narratives provided by respondents and how they were related to the major quantitative takeaways from the study. This portion of the study serves to explore correlations that may exist but cannot be extrapolated to prove causality.

Similarly, this research is unable to rule out the presence of other confounding variables that drive the relationships observed in the quantitative analyses. The leading sources of omitted variable bias are controlled for but it is entirely possible that other factors exist. As a result, this study is not able to definitively prove causality between variables, only correlations. Furthermore, these findings are not necessarily externally valid beyond the United States. This research intentionally zeroed in on the American public given a lack of existing data and the opportunity that the scope of the domestic response within the United States presented.

Finally, characteristics of the pandemic environment itself are limiting to the generalizability of these findings. COVID-19 was a very specific, novel pathogen. Compared to other contagions, it possessed a relatively low fatality rate. The perceptions elicited by this virus may be different than that of other viruses or other classifications of health emergency or natural disaster. This study did not consider other SARS outbreaks, other diseases, or responses past or present in any country aside from the United States.

## 5 Conclusions

With national-level crises requiring or warranting the deployment of armed actors within states observed ever-increasingly, the field of humanitarian-military relations remains a relevant field of study under the broader umbrella of civil-military research. Specifically, the pronounced lack of scholarship addressing affected population sentiment toward military and law enforcement responses, both in foreign intervention and domestic response settings, presents a challenge to optimizing aid but an opportunity for further scholarship. In an effort to begin closing the gap in understanding, this research takes advantage of the large-scale mobilization of military and law enforcement actors during the COVID-19 pandemic in the United States to study the resulting perceptions harbored by the American public.

While unquestionably context-specific and dependent upon the unique features of a highly contentious and wide-reaching public health emergency, the survey administered yielded a number of insights into how the general public regards military and police actors in such settings. Majorities agreed with the necessity of involving armed actors, that the quality of the respective contributions were positive, and acknowledged comfort with the ways in which military and police were utilized. However, military personnel earned higher favorability and were the subjects of less trepidation across the board when compared to law enforcement, preferred by a higher proportion of participants to be involved in future national emergencies.

These findings are nuanced by the fact that the military historically has enjoyed the most trust of any government institution in the United States–a trend that is not necessarily true around the world–and that it fulfilled roles that more closely resembled those of civilian healthcare workers because of comparable capabilities that law enforcement lacked. Moreover, civilian healthcare workers were found to be preferred in pandemic response and earned the highest marks of trust when offered in parallel with armed actors, but these feelings did not preclude participants from still supporting the future involvement of armed actors as a supporting element to civilian-led responses. Highly influential in shaping the perceptions held by respondents were factors such as vaccination status, political party affiliation, political ideology, gender, and age, found to be correlated with views even when controlling for the frequent overlap between these characteristics and other demographic variables.

Despite the notion embedded in numerous response frameworks that military resources are only to be used as a last resort, their unique capacity and prevailing public sentiment may warrant shaping public health policy to better integrate their capabilities into future responses and lower the bar for entry to optimize the quality of care provided to affected populations. Ever-important is clear, factually-accurate, and repetitive engagement with the media and the public to alleviate concerns of the politicization of apolitical armed entities and the strong-arming of what should remain civilian-led efforts. There remains vast opportunity to examine how affected population sentiment may evolve in other emergency settings–whether in different areas of the world or in events outside of public health and pandemic aid–as the political environment studied within this research was distinct and polarized in ways that other disaster settings may not be.

## Data Availability

The raw data supporting the conclusions of this article will be made available by the authors, without undue reservation.

## Footnotes

^1^Throughout the remainder of this manuscript, “armed actor” is used interchangeably with “the United States military and law enforcement agents” for the sake of brevity. Such a characterization is consistent with UN convention. However, it is worth noting that the term also encompasses various militarized non-state entities found in international settings, which were not relevant in the United States during the COVID-19 pandemic.

^2^Other relevant guidelines include the 2006 revision of the Guidelines On The Use of Military and Civil Defence Assets To Support United Nations Humanitarian Activities in Complex Emergencies, found here. While the Oslo Guidelines are international and explicitly apply to public health settings, this document serves as a counterpart that provides additional instruction for UN forces in international emergencies and humanitarian efforts complicated by an armed conflict element, and, “may also be of value in other large-scale emergencies” (p. 5).
