# Supplementary figures and images for: Gauging public perceptions of military and police roles in US domestic pandemic response during COVID-19
Source: Front Public Health. 2025 Jun 18;13:1569263. doi: 10.3389/fpubh.2025.1569263 (PMC12217939; doi:10.3389/fpubh.2025.1569263)

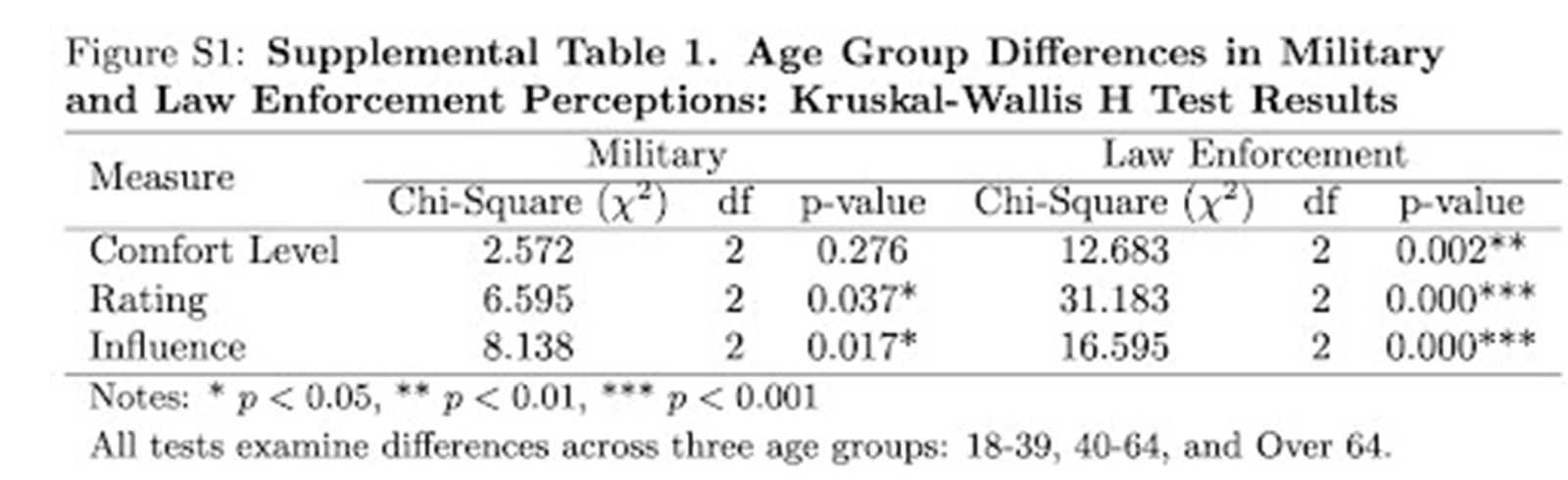

Supplement: Supplementary file 1 [file Image_1.jpeg]

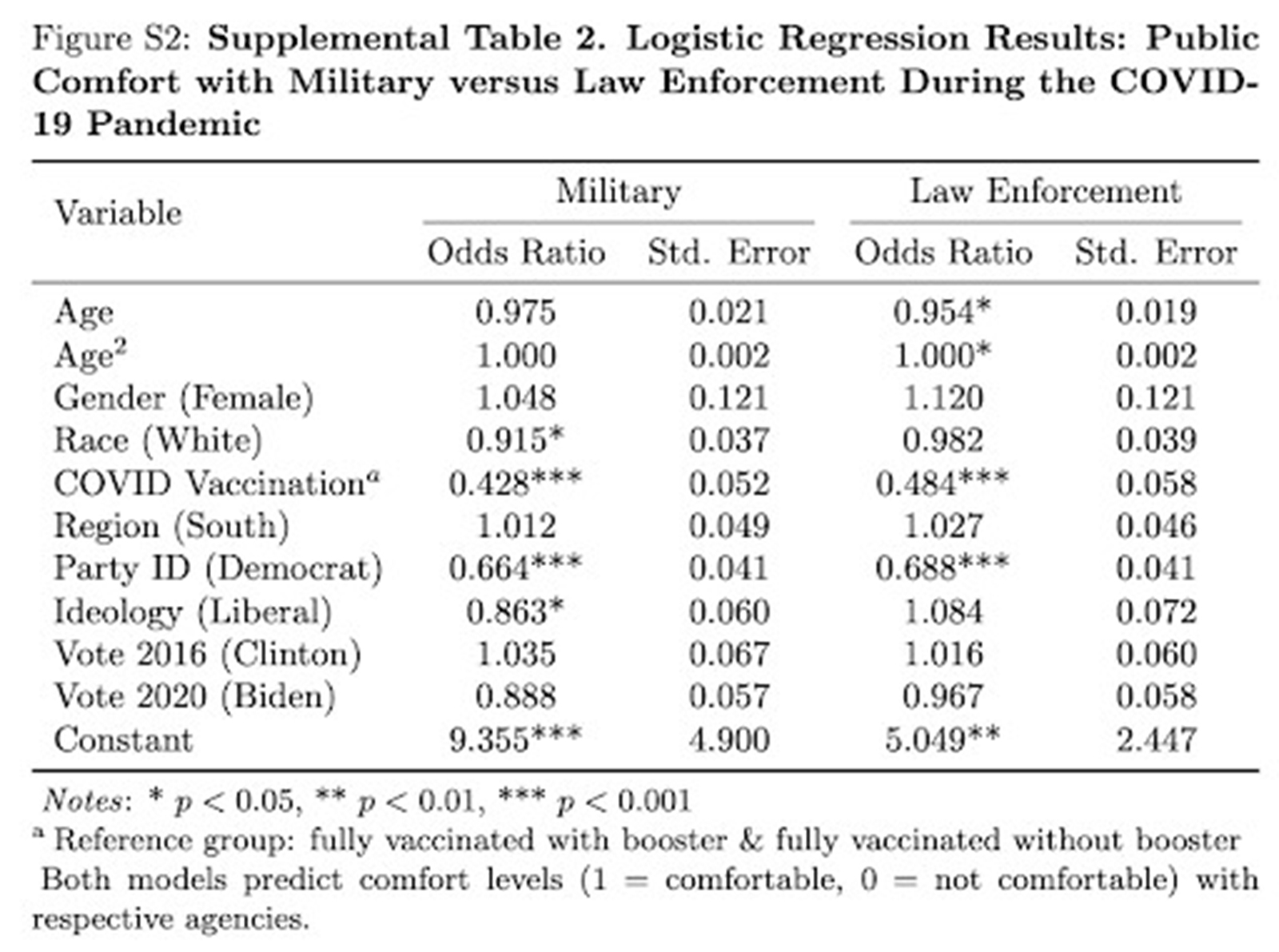

Supplement: Supplementary file 2 [file Image_2.jpeg]

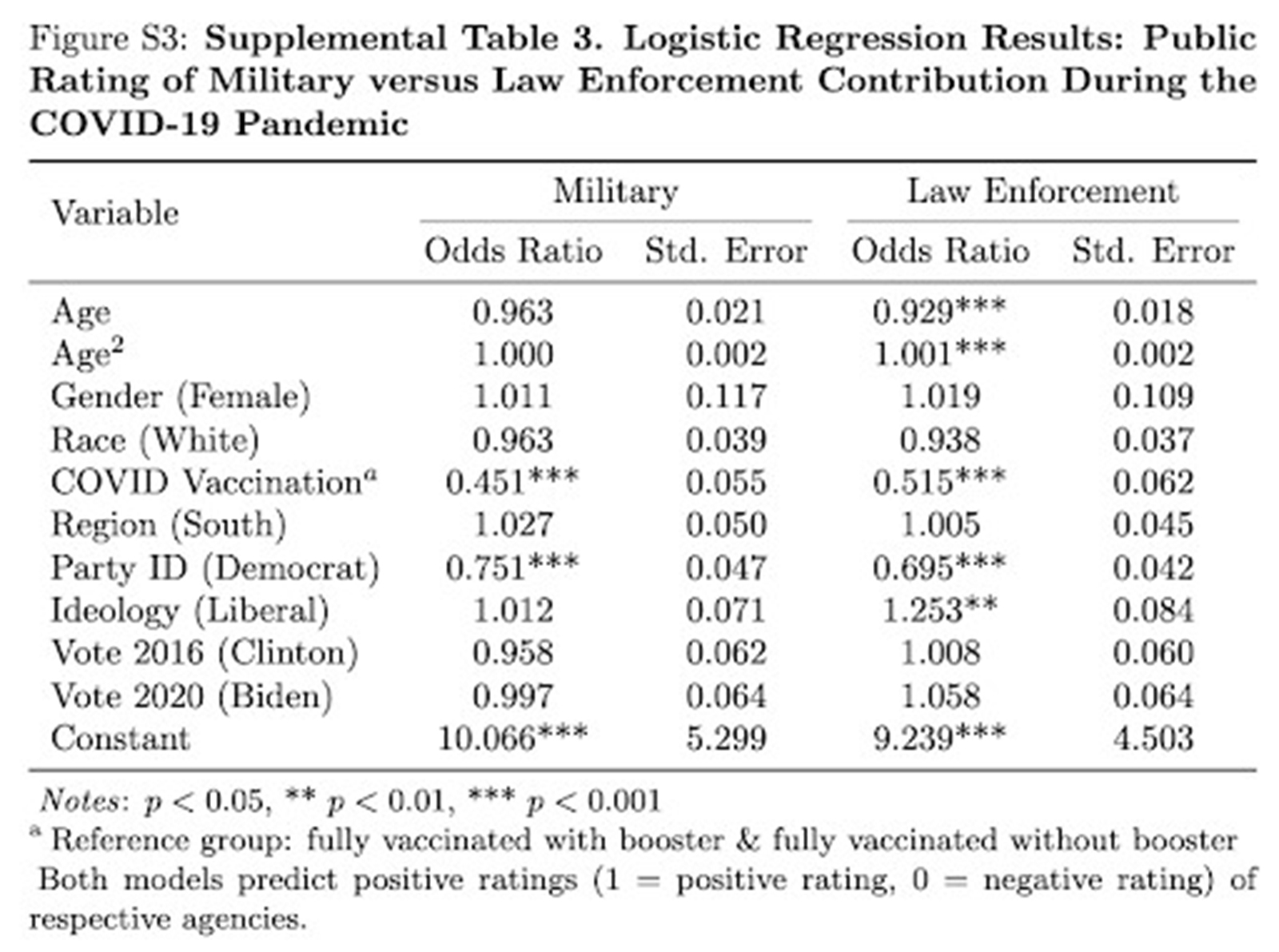

Supplement: Supplementary file 3 [file Image_3.jpeg]

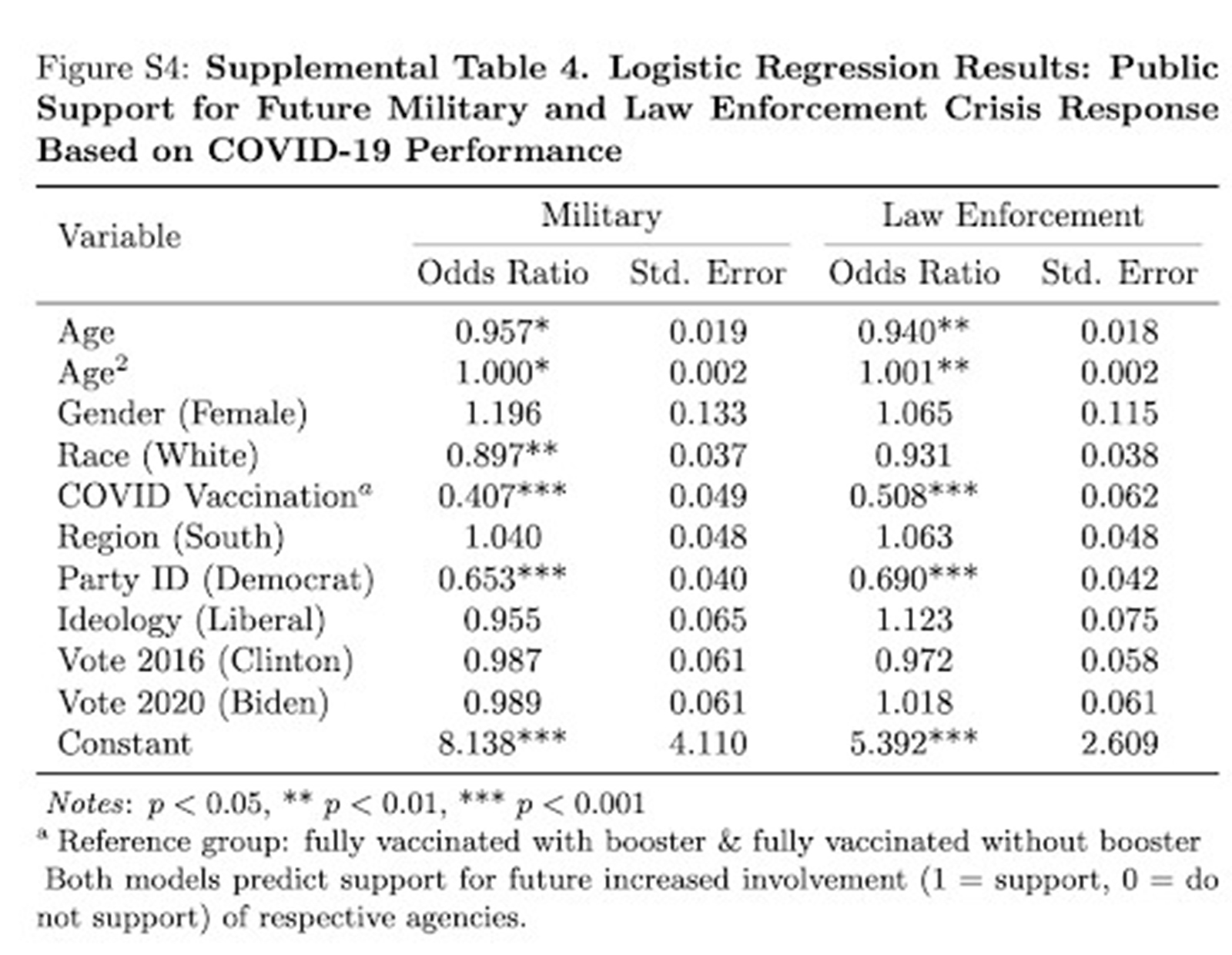

Supplement: Supplementary file 4 [file Image_4.jpeg]

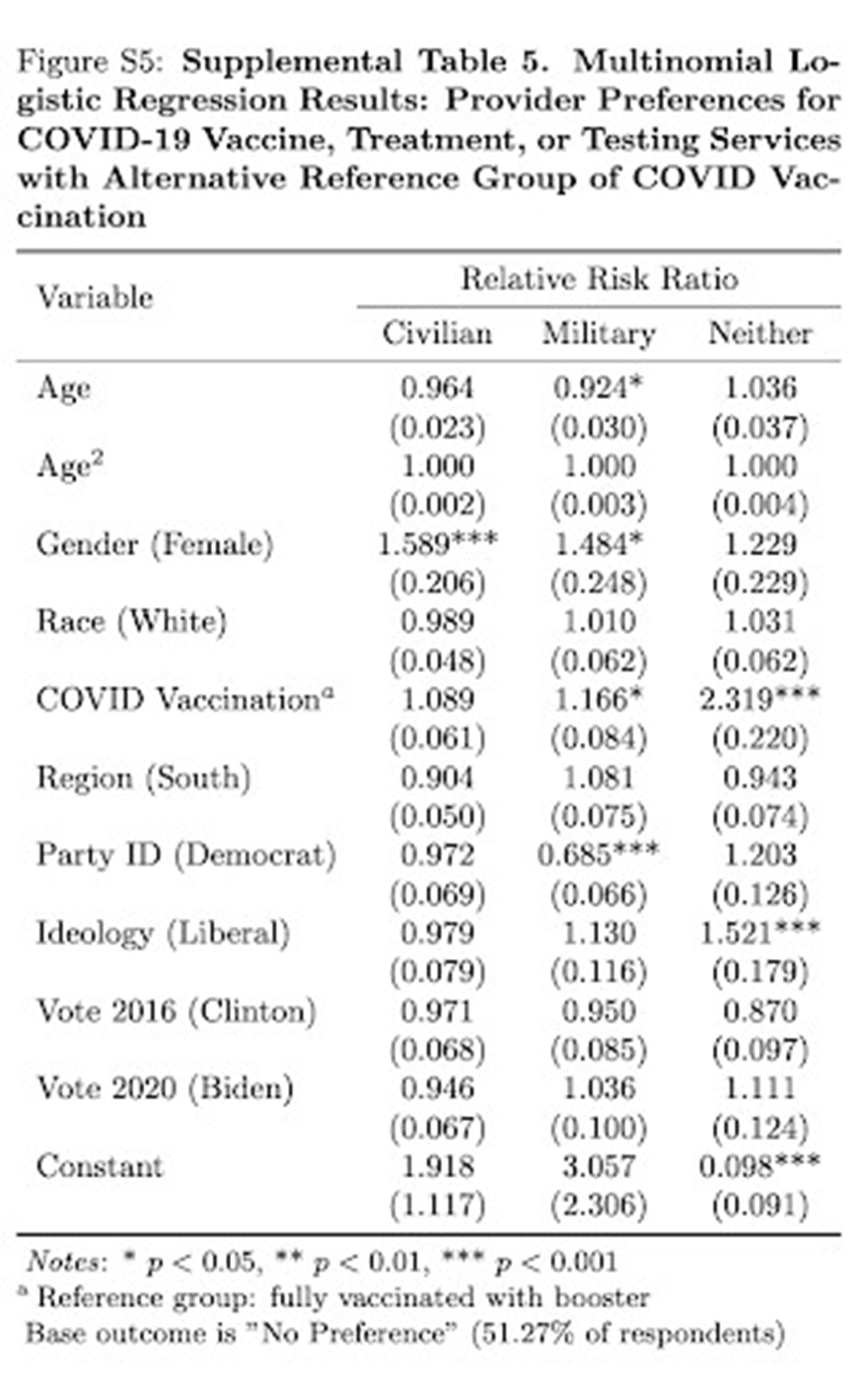

Supplement: Supplementary file 5 [file Image_5.jpeg]

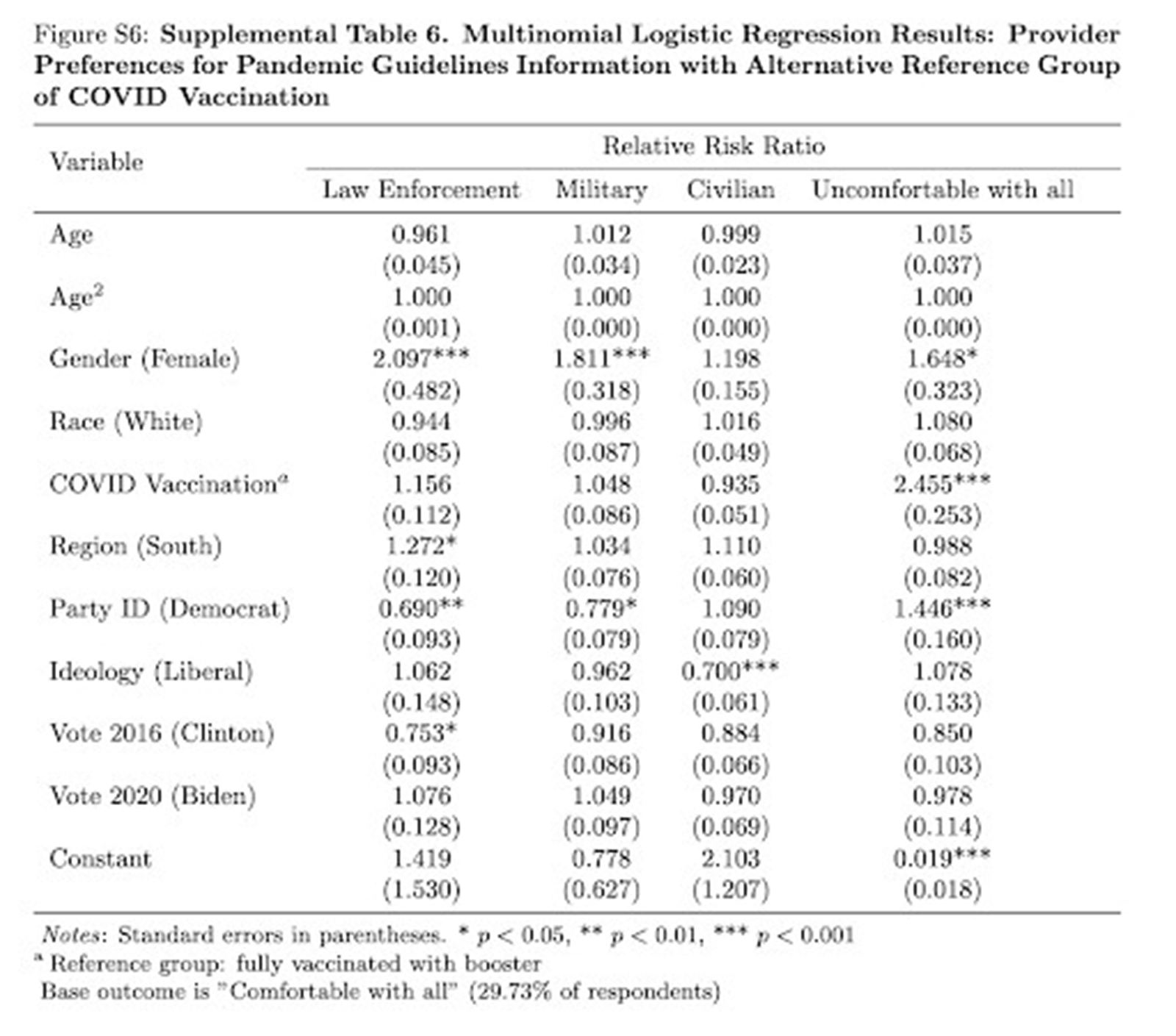

Supplement: Supplementary file 6 [file Image_6.jpeg]

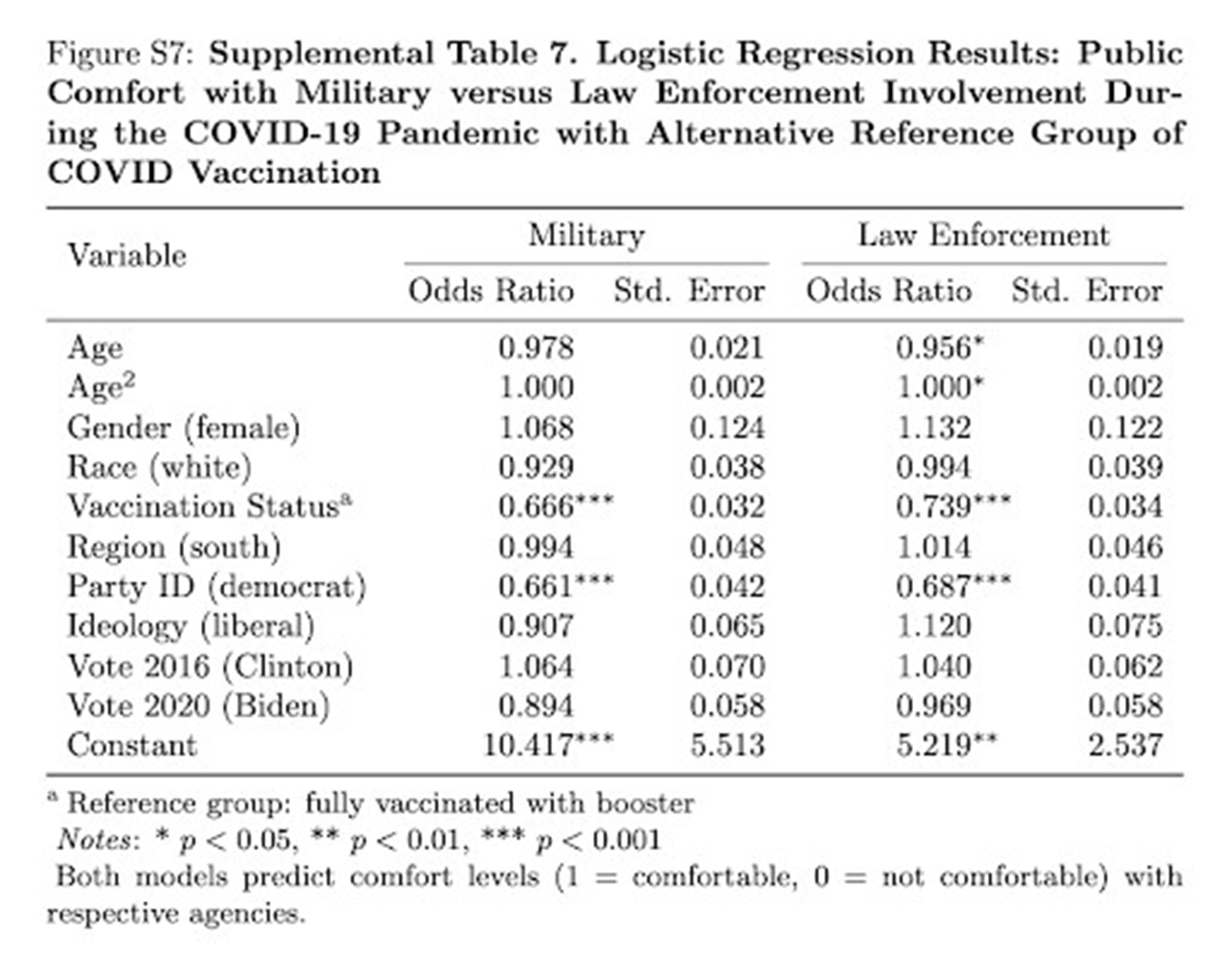

Supplement: Supplementary file 7 [file Image_7.jpeg]

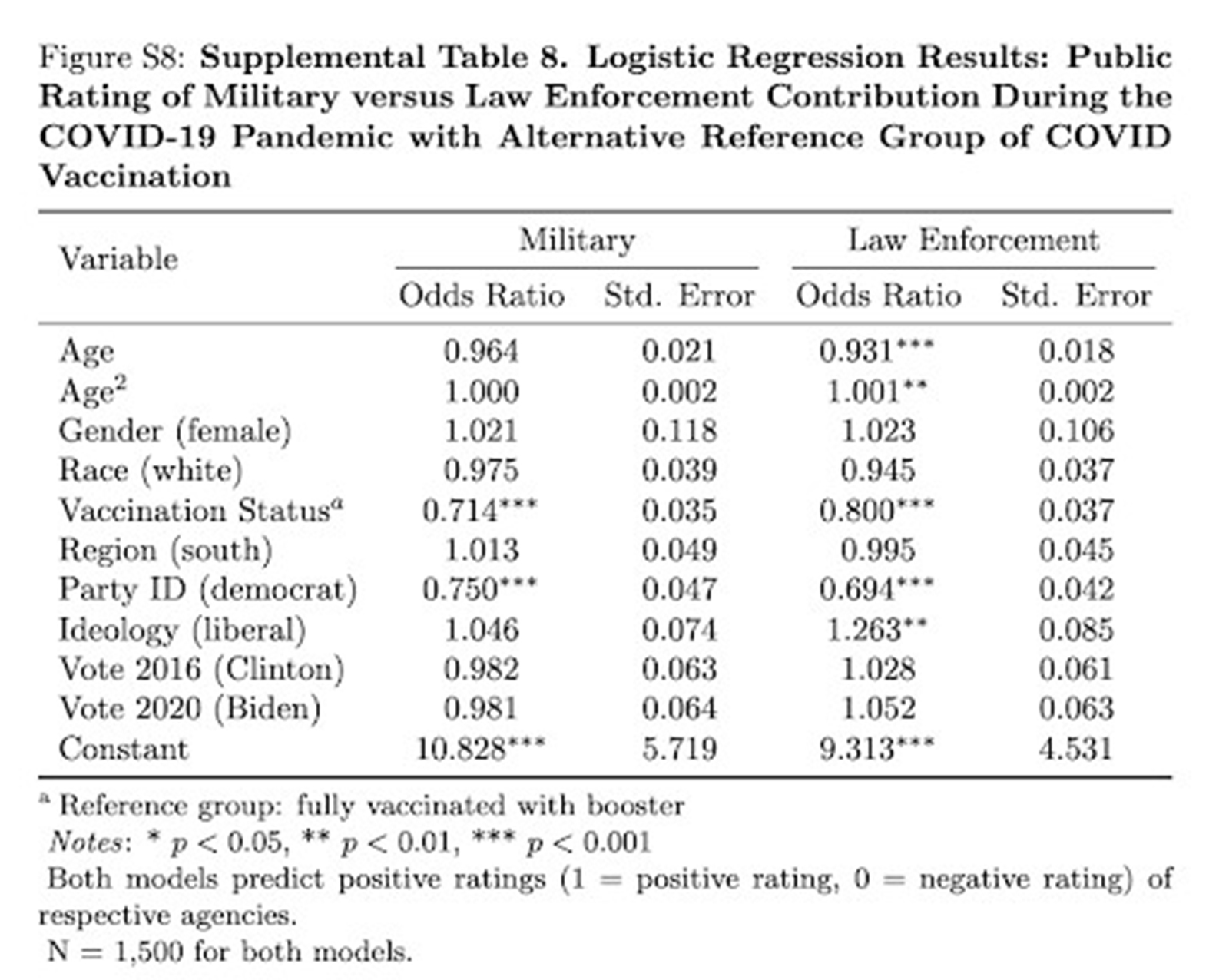

Supplement: Supplementary file 8 [file Image_8.jpeg]

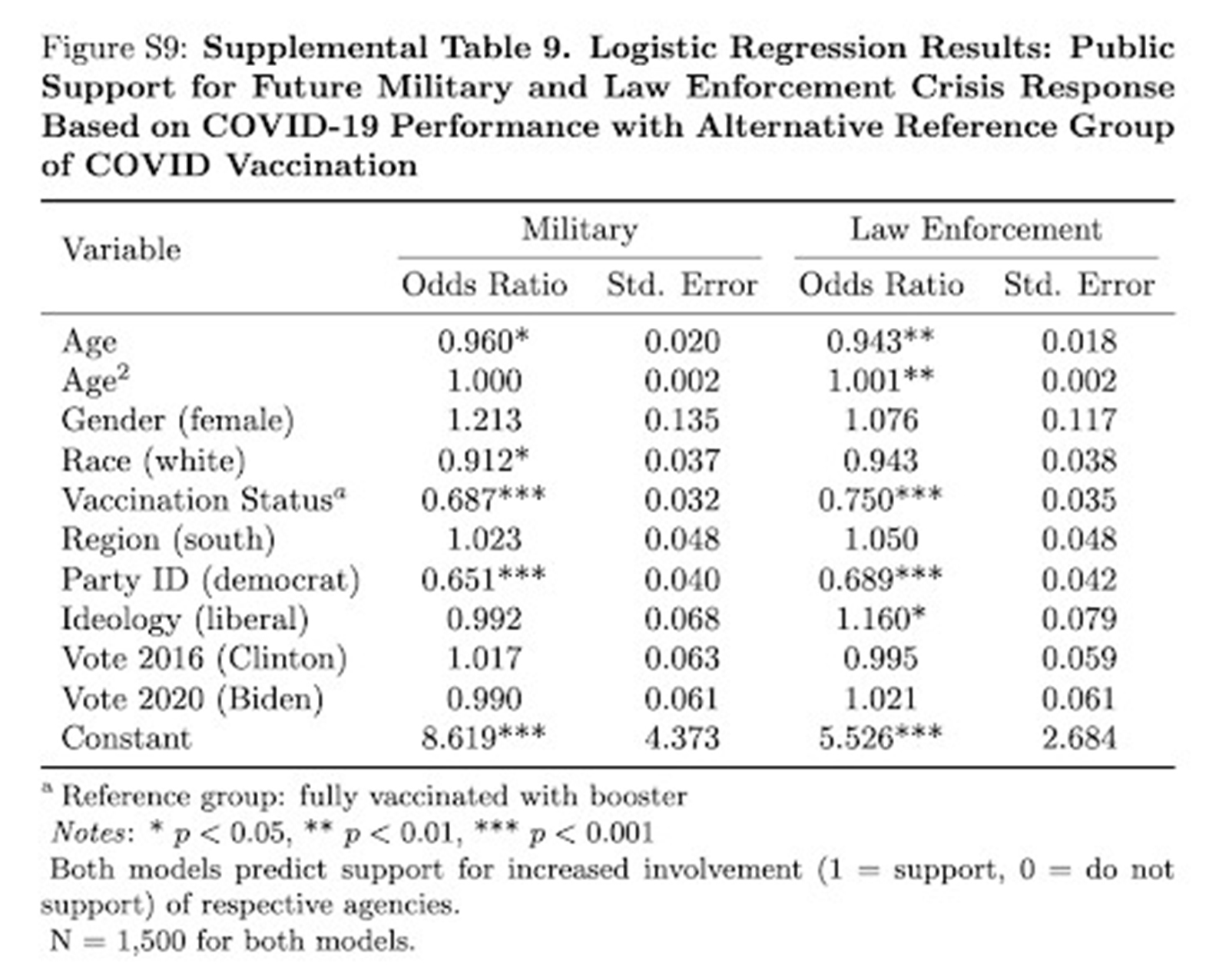

Supplement: Supplementary file 9 [file Image_9.jpeg]
